# Structural basis for the recognition of human hemoglobin by the heme-acquisition protein Shr from *Streptococcus pyogenes*

**DOI:** 10.1038/s41598-024-55734-x

**Published:** 2024-03-05

**Authors:** Akinobu Senoo, Masato Hoshino, Toshiki Shiomi, Makoto Nakakido, Satoru Nagatoishi, Daisuke Kuroda, Ichiro Nakagawa, Jeremy R. H. Tame, Jose M. M. Caaveiro, Kouhei Tsumoto

**Affiliations:** 1 Laboratory of Protein Drug Discovery, Graduate School of Pharmaceutical Sciences, Kyushu University, 3-1-1 Maidashi, Higashi-ku, Fukuoka City, 812-8582 Japan; 2 Department of Chemistry and Biotechnology, School of Engineering, The University of Tokyo, 7-3-1 Hongo, Bunkyo-ku, Tokyo, 113-8656 Japan; 3 Department of Bioengineering, School of Engineering, The University of Tokyo, 7-3-1 Hongo, Bunkyo-ku, Tokyo, 113-8656 Japan; 4 Medical Device Development and Regulation Research Center, School of Engineering, The University of Tokyo, 7-3-1 Hongo, Bunkyo-ku, Tokyo, 113-8656 Japan; 5 Research Center for Drug and Vaccine Development, National Institute of Infectious Diseases, 1-23-1 Toyama, Shinjuku-ku, Tokyo, 162-8640 Japan; 6 Department of Microbiology, Graduate School of Medicine, Kyoto University, Yoshida-Konoe-cho, Sakyo-ku, Kyoto, 606-8501 Japan; 7 Drug Design Laboratory, Graduate School of Medical Life Science, Yokohama City University, 1-7-29 Suehiro, Yokohama, Kanagawa 230-0045 Japan; 8 The Institute of Medical Sciences, The University of Tokyo, 4-6-1 Shirokanedai, Minato-ku, Tokyo, 108-8629 Japan

**Keywords:** Hemoglobin, *Streptococcus pyogenes*, Protein–protein interaction, Heme, Host–pathogen interaction, X-ray crystallography, Surface plasmon resonance, MD simulations, Heme acquisition, Biochemistry, X-ray crystallography, Biophysics, Permeation and transport, Drug discovery, Target identification, Microbiology, Pathogens, Structural biology, X-ray crystallography

## Abstract

In Gram-positive bacteria, sophisticated machineries to acquire the heme group of hemoglobin (Hb) have evolved to extract the precious iron atom contained in it. In the human pathogen *Streptococcus pyogenes*, the Shr protein is a key component of this machinery. Herein we present the crystal structure of hemoglobin-interacting domain 2 (HID2) of Shr bound to Hb. HID2 interacts with both, the protein and heme portions of Hb, explaining the specificity of HID2 for the heme-bound form of Hb, but not its heme-depleted form. Further mutational analysis shows little tolerance of HID2 to interfacial mutations, suggesting that its interaction surface with Hb could be a suitable candidate to develop efficient inhibitors abrogating the binding of Shr to Hb.

## Introduction

Competition between pathogens and host organisms for critical molecular entities is a matter of life and death^1^. Living in a competitive and in many cases resource-poor environment, bacteria are endowed with a rich variety of molecular systems to efficiently acquire nutrients from the host organism. For example, Gram-positive human pathogens *Staphylococcus aureus* and *Streptococcus pyogenes* have dedicated protein machineries to acquire the precious iron atom contained in the heme group of hemoglobin (Hb)^2–4^. The most intensively studied heme-acquisition machinery is that of *S. aureus*, termed the iron-regulated surface determinant (Isd)^5^. The Isd machinery comprises 12 proteins that extract, transport, and metabolize the heme group from Hb to obtain the valuable iron atom contained in it.

Similarly, *S. pyogenes* possesses the *sia* gene cluster encoding proteins that form a distinct heme-transfer system from that of Isd^6^. One of the key components of the heme-acquisition system in *S. pyogenes* is the extracellular Shr protein, a large polypeptide comprising 1,275 residues that acquires heme from Hb and transfers it to downstream proteins^7,8^. Indeed, the acquisition of heme, and specially of the iron atom contained in it by Shr is a key element for the full virulence of *S. pyogenes*^9^. Structurally, Shr is composed of two distinctive hemoglobin-interacting domains (HID1 and HID2), two NEAr Transporter (NEAT) domains separated by a leucine-rich repeat (LRR) domain, and a linker domain connecting the HID2 domain with the NEAT1 domain^10,11^.

Biophysical characterization of the N-terminal region (NTD), comprising HID1 and HID2, demonstrated its interaction with hemoglobin (Hb) by two independent techniques (surface plasmon resonance (SPR) and isothermal titration calorimetry (ITC))^12^. A follow-up study employing each individual domain HID1 and HID2 showed a preferential binding of HID2 to Hb with respect to that of HID1^11^. These results suggested a more prominent role of HID2 during heme acquisition by Shr. Indeed, a recent report of the crystal structures of HID2 bound to Hb suggested a different molecular mechanism for heme extraction by Shr from *S. pyogenes* with respect to that of other bacterial systems studied so far^13^.

Herein we sought to characterize the recognition of Hb by the HID2 receptor of Shr from *S. pyogenes* using structural and biophysical techniques. The structure of the HID2-Hb complex revealed that HID2 binds α- and β-chains of Hb resulting in an architecture in which HID2 employs both Hb and heme to engage its target, in general agreement with that of a structure recently reported, although with a much greater HID2:Hb stoichiometry. Site-directed mutagenesis and molecular dynamics (MD) simulations revealed key features of the protein–protein interaction surface and recognition mechanism. Together with the structural information, some possible drug modalities to inhibit Shr-Hb complex are suggested from this work.

## Results and discussion

### Structure of HID2 in complex with human Hb

**Figure 1:**
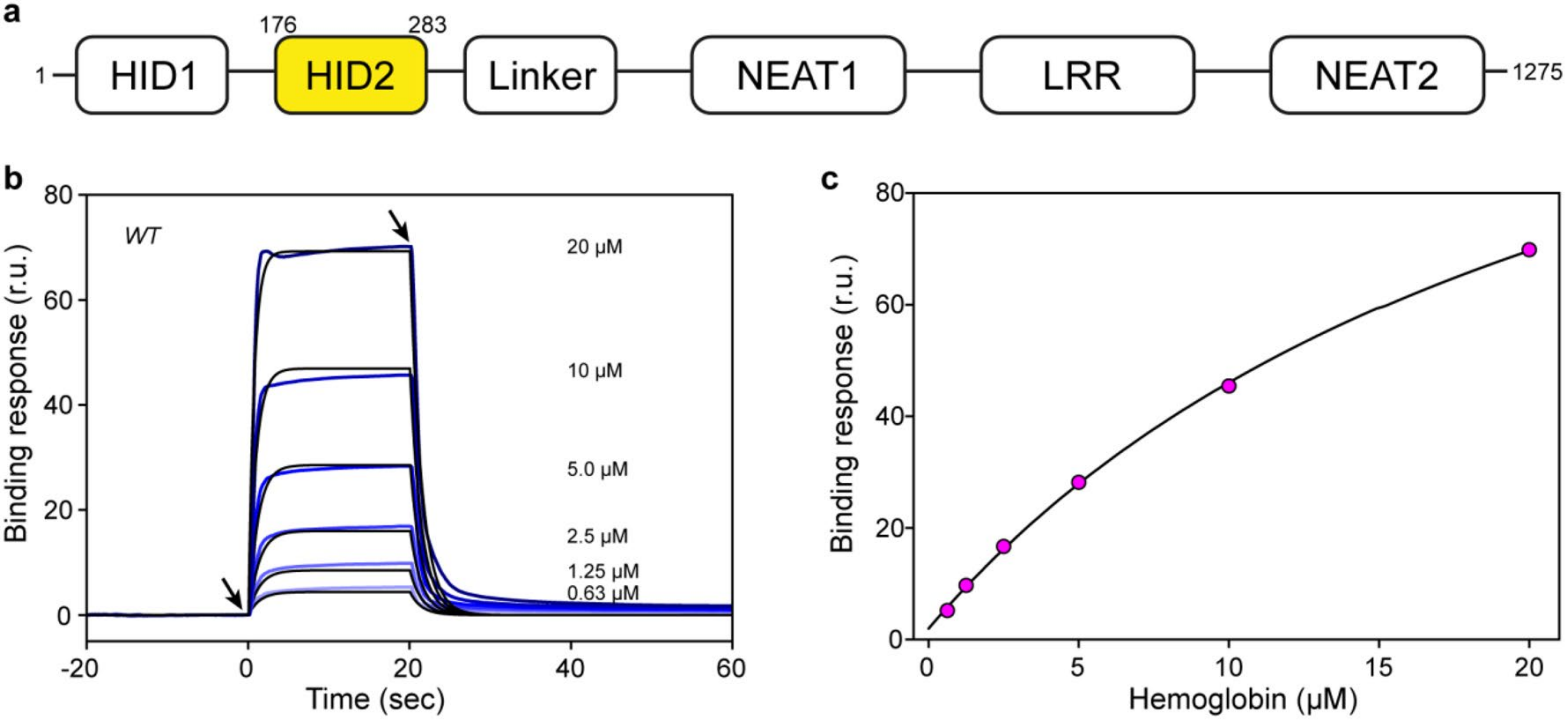
Binding of HID2 to human Hb. (**a**) Primary structure of Shr. The long sequence of Shr is divided into several domains. The domain HID2 examined in the current study is highlighted in yellow. (**b**) Sensorgrams (blue) corresponding to the binding of human Hb (analyte) to a surface decorated with HID2. The arrows on the left and right indicated the beginning and end of addition of analyte to the solution flowing through the sensor chip. The concentration of human Hb employed in each binding experiment is indicated in the panel. Black traces corresponded to the best fitting to the experimental data calculated with the software BiaEvaluation. (**c**) Binding affinity was also determined from the maximal binding response in equilibrium at each concentration of human Hb employed.

Recombinant HID2 comprising residues 169–294 was purified to homogeneity and its binding to human Hb examined by SPR (Fig. 1, Table 1). The dissociation constant (*K*_*D*_) determined from the analysis of the kinetic data was 22 μM. The association rate constant (*k*_*on*_ = 3.0 × 10^4^ M^−1^ s^−1^) was relatively slow, whereas the dissociation rate constant (*k*_*off*_ = 0.65 s^−1^) was fast, describing a box-shaped sensorgram. When the analysis was performed using the maximal response in equilibrium a value of 25 μM was obtained, which falls within 15% of the value determined by the kinetic analysis. Both these values are comparable to a previous study employing a similar construct^11^.

**Table 1:** Binding affinity between human Hb and HID2 mutants.

Protein	*k*_*on*_ (M^−1^ s^−1^)	*k*_*off*_ (s^−1^)	*K*_*D*_ (μM)^a^	*Affinity loss*
WT	30,000 ± 160	0.65 ± 0.025	22 ± 0.12	1
R196A	n.d.^b^	n.d	n.d	> 1000-fold
Y197A	n.d	n.d	n.d	> 1000-fold
Q209A	1800 ± 4.3	3.0 ± 0.058	1600 ± 37	73-fold
I224A	620 ± 2.3	4.9 ± 0.15	7900 ± 290	360-fold
S225A	n.d	n.d	n.d	> 1000-fold
M238A	n.d	n.d	n.d	> 1000-fold

^a^Values obtained from the kinetic constant.

^b^n.d.: binding not detected.

**Figure 2:**
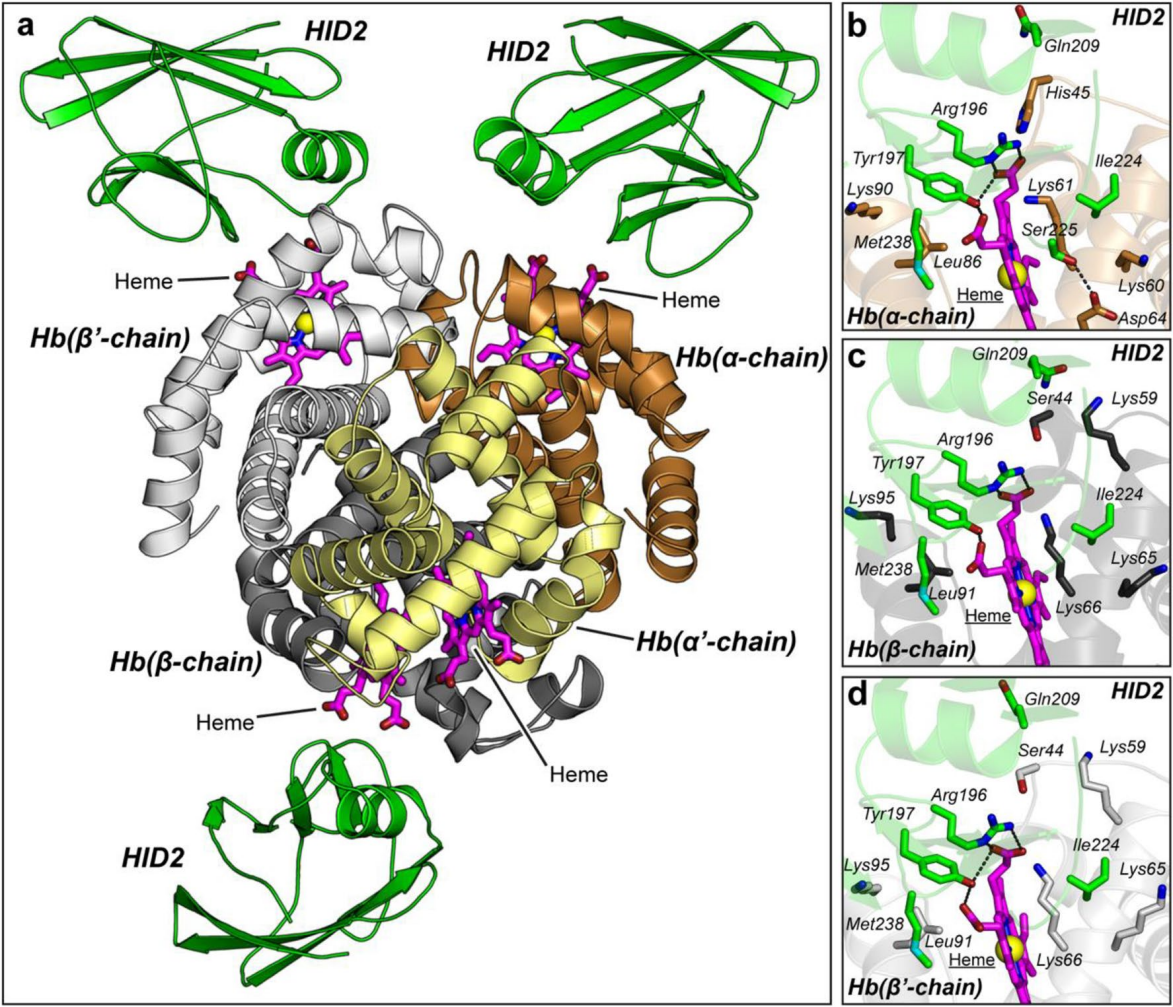
Crystal structure of HID2 bound to human Hb. (**a**) Overall structure. Three molecules of HID2 appeared bound immediately in front of the heme group of tetrameric human Hb. The four chains of hemoglobin (two α and two β) are shown in orange/yellow and dark/light gray. HID2 is depicted in green. The heme group is shown as magenta sticks. (**b**) Close-up view of the interaction between HID2 and chain α of human Hb. Residues of HID2 interacting with residues of human Hb as determined by the PISA software are depicted as green and orange sticks, respectively. The heme group, also participating in the interaction is shown as magenta sticks. H-bonds (distance ≤ 3.3 Å) between HID2 and human Hb including heme are shown as broken black lines. Close-up view of the interaction between HID2 and (**c**) chain ß (dark gray) or (**d**) chain ß’ (light gray) of human Hb.

To understand the structural basis of the interaction between HID2 and human hemoglobin, we next obtained the crystal structure of the complex at a resolution of 2.75 Å (Fig. 2, Table 2). The asymmetric unit in the crystal contained the intact tetramer of Hb, comprising two α- and two β-chains in a conformation consistent with that of the relaxed state. Each chain of hemoglobin displayed the characteristic heme group coordinated to the proximal His residue and a water molecule that was modeled in the distal coordination position (Figure S1). Three molecules of HID2 were bound to a tetrameric unit of Hb: one molecule of HID2 to one of the two α-chains, and one molecule of HID2 to each of the two β-chains (overall 75% occupancy). We believe the stoichiometry Hb: HID2 (1:3) may reflect a particular obstacle of the complex to crystallize in the expected 1:4 complex because of unfavorable crystal contacts (Figure S2). The stoichiometry found in this crystal structure contrasts to that observed in another structure of the complex, in which only three molecules of HID2 were bound to two tetramers of Hb in the asymmetric unit (37.5% occupancy)^13^.

**Table 2:** Data collection and refinement statistics.

	HID2	Hb-HID2 complex
Data collection		
Space group	P 6_3_	C 2
Unit cell		
a, b, c (Å)	76.1, 76.1, 33.9	178.5, 52.8, 129.2
α, β, γ (°)	90.0, 90.0, 120.0	90.0, 118.4, 90.0
Resolution (Å)	32.9–1.80 (1.90–1.80)	44.2–2.75 (2.90–2.75)
Wavelength	1.0000	1.0000
Observations	114,672 (16,365)	114,191 (11,426)
Unique reflections	10,571 (1545)	24,514 (3018)
*R*_*merge*_	0.133 (0.524)	0.110 (0.582)
*R*_*p.i.m*_	0.042 (0.167)	0.056 (0.326)
CC_1/2_	0.997 (0.907)	0.991 (0.749)
*I / σ (I)*	14.5 (4.6)	8.7 (2.0)
Multiplicity	10.8 (10.6)	4.7 (3.8)
Completeness (%)	99.8 (99.4)	87.9 (75.1)
Refinement statistics		
Resolution (Å)	32.9–1.80	44.2–2.75
*R*_*work*_ / *R*_*free*_ (%)	15.5/20.6	21.2/25.9
No. protein chains	1	4 (Hb) + 3 (HID2)
No. atoms		
HID2	912	2505
Hemoglobin	–	4384
Heme	–	142
Other	1	10
Water	114	22
B-factor (Å^2^)		
HID2	16.1	85.5
Hemoglobin	–	59.5
Heme	–	51.1
Others	33.6	74.3
Water	23.3	40.5
Ramachandran plot		
Preferred (%)	88.6	86.9
Allowed (%)	10.5	12.7
Outliers (%)	1.0	0.4
RMSD Bond (Å)	0.013	0.006
RMSD Angle (°)	1.71	1.52
PDB	7CUD	7CUE

Statistical values given in parenthesis refer to the highest resolution bin.

The conformation of HID2 did not appreciably change among the three molecules in the asymmetric unit, achieving an overall root mean square deviation (RMSD) of less than 0.6 Å for the α-carbon of the main chain. No significant structural changes in HID2 bound to the β-chain of Hb are observed with respect to that of HID2 bound to the α-chain (RMSD < 0.5 Å). Similarly, the structure of HID2 was nearly indistinguishable from that of the unbound protein also determined herein (RMSD 0.6 Å, Figure S3) or to the previously reported structure of a slightly shorter construct (PDB entry code 6DKQ, RMSD = 0.56 Å)^11^ or to the previously reported structure of HID2 in complex with Hb (PDB entry code 8DOV, RMSD < 0.3 Å)^13^. These results indicate that HID2 does not undergo significant conformational changes to recognize and stably bind to the target region in Hb, suggesting a mechanism of lock-and-key similar to that observed for some VHH antibodies (nanobodies) binding to their antigens^14^.

### Analysis of the interaction surface

The interaction surface between HID2 and the proteinaceous region of the α-chain and the β-chain of Hb is small, and of similar extent (904 and 885 Å^2^, respectively) (Table S1). These values are below the theoretical minimum surface area required for productive protein–protein binding (> 1000 Å^2^)^15,16^. Among the residues at the interface, aromatic or hydrophobic residues such as Tyr197, Ile224 or Met238 from HID2 contributed most to the buried surface area (BSA). In contrast, from the α- or β-chain of Hb, the presence of three Lys residues was predominant: three Lys residues from the α-chain and four Lys residues from the β-chain, although only two of them (Lys 60 of α-chain and Lys95 of the β-chain) engaged in salt bridges with residues of HID2 (distance < 4.5 Å, Table S1, Table S2). These observations were similar to those made in the previous structure of the complex (PDB entry code: 8DOV)^13^. Additional polar interactions between the interacting proteins were scarce, and indeed only two H-bond (< 3.3 Å) were found, specifically between side chain of Asp64 of α-chain and Ser225 of HID2, and between the backbone oxygen of Ser44 and the side chain of Gln209 of HID2 (Fig. 2, Table S2). It is noteworthy that the later interaction between Ser225 of HID2 is specific of interface with the α-chain of Hb, whereas the interaction involving Gln209 of HID2 is specific in the interaction with β-chain of Hb. In addition, the previously reported crystal structure also indicated the presence of water molecules at the interface between the E-helix of Hb and several residues of HID2 that could support the primary interactions enumerated above^13^. Our structure was determined at lower resolution and no evidence of interfacial waters was found.

The binding interface between HID2 and Hb was significantly increased when the contact interface between HID2 and heme is included. A total of ~ 280 Å^2^ of buried surface and several H-bonds are contributed to the overall binding between HID2 and Hb when the heme prosthetic group is included (Table S2, Table S3). The binding surface with the α-chain increases to 1,184 Å^2^, and that with the β-chain to 1,171 Å^2^, and the number of H-bonds increases by an average of three new bonds for each HID2-Hb chain complex (Table S2). Of special importance is the role of the dyad of residues Arg196-Tyr197 of HID2, which engage in several H-bonds with the propionate group of the heme group. These interactions with heme may explain why HID2 specifically recognizes the holo form of Hb^10^, although they do not explain how heme molecules of Hb are extracted by Shr. The heme-mediated binding mechanism ensures effective engagement of Shr to Hb molecules loaded with the heme group, thus giving rise to productive acquisition of heme in each binding event. The binding of HID2 did not result in significant conformational changes of Hb. For example, the RMSD between the α-chains (one of them displaying HID2 bound) was only 0.35 Å. It is reasonable to think that other regions of Shr could assist in heme extraction, as suggested in a recent study implicating HID1 in heme extraction by a “cap and release” mechanism^13^.

**Figure 3:**
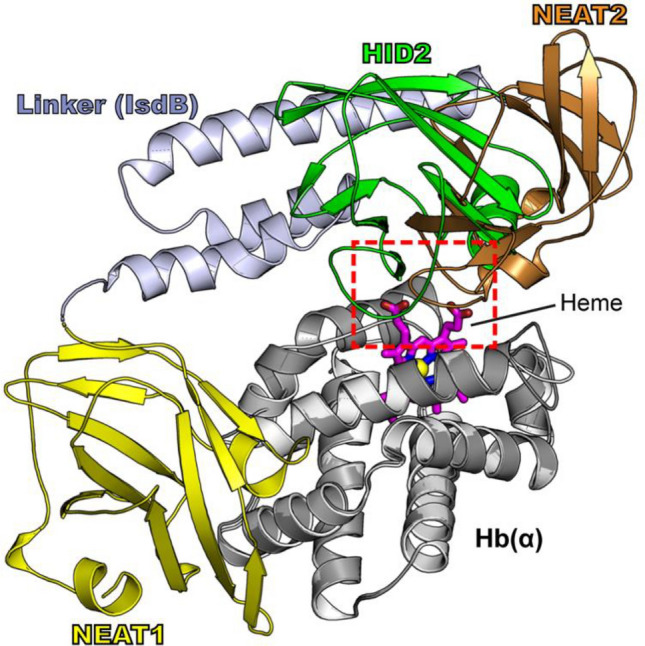
Binding architecture. Superposition of the structures of HID2 and IsdB from *S. aureus* each bound to human Hb. HID2 (green) binds to a region of human Hb that largely overlaps (red box) with that of IsdB-NEAT2 (a heme extracting domain) and partially with to that of the linker of IsdB, but does not coincide with that of NEAT1 (the Hb binding domain of IsdB). The heme group is depicted with magenta sticks. For simplicity, only the heme bound to the molecule of Hb in complex with HID2 is shown.

The role of the heme group for the efficient binding of HID2 to Hb is a unique feature of Shr when compared to other well-characterized heme-acquisition proteins such as IsdH or IsdB (Fig. 3)^17,18^. The proteins IsdH and IsdB from *S. aureus* employ specific NEAT domains to bind to regions of Hb that do not interfere with the movement of heme to reach the heme binding modules (NEAT2 of IsdB or NEAT3 of IsdH). In the structure presented in this work, HID2 is located in the position occupied by the heme binding domain NEAT2 of IsdB, i.e. at the entrance of the heme binding pocket of Hb.

### Site directed mutagenesis

Site-directed mutagenesis combined with SPR were employed to evaluate the relative importance of various residues of HID2 located at the interaction surface with Hb (Figure S4 and Table 1). All six residues were selected based on their location in the contact interface of Shr with Hb, and each one was mutated to Alanine (Ala). The purified Ala-mutants of HID2 were expressed successfully and showed an essentially identical secondary structure to that of the wild-type protein (Figure S5). Importantly, all mutants showed little or no binding to Hb, indicating the importance of these positions in the wild-type (WT) for binding. Compared with WT HID2, all the mutants resulted in significantly lower affinity for Hb, ranging from 75-fold decrease in Q209A to the complete abrogation of binding in R196A, in Y197A, in S225A, and in M328A. The affinity of I224A for Hb was reduced by 360-fold compared with WT HID2. The mutations disrupted the association step (*k*_*on*_) and accelerated the unbinding (*k*_*off*_) event. Structurally, these residues contributed to the interaction in various ways.

**Figure 4:**
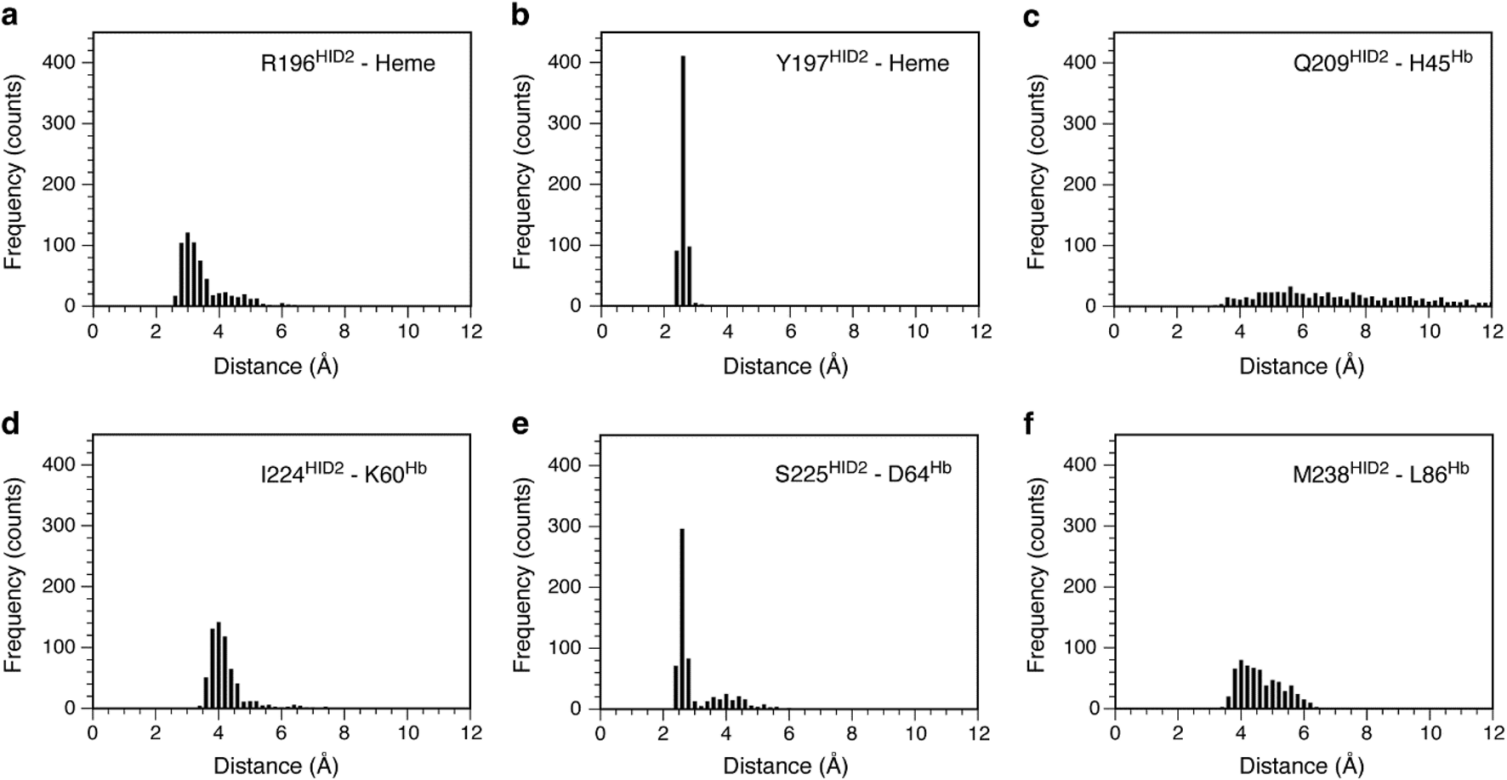
Frequency histogram of interactions at interface of HID2-Hb. Distances between (**a**) HID2 Arg196 (NE) and heme (O1D), (**b**) HID2 Tyr197 (OH) and heme (O1A or O2A), (**c**) HID2 Gln209 (NE2) and α-chain of Hb His45 (CB), (**d**) HID2 Ile224 (CG2) and α-chain of Hb Lys60 (CB), (**e**) HID2 Ser225 (OG) and α-chain of Hb Asp64 (OD2), (**f**) HID2 Met238 (CB) and α-chain of Hb Leu86 (CD2) are shown as frequency histograms.

To strengthen the mutation analysis above, molecular dynamics (MD) simulations were performed using the coordinates of the Hb tetramer in complex with one of the three HID2 molecules, i.e. that bound to the α-chain of Hb as a starting structure. A previous binding study employing ITC^11^ and our own observation that no major conformational changes occur (low RMSD differences between bound/unbound HID2) suggests that the binding of one HID2 molecule to either chain of Hb will not influence the binding of the next HID2 to the next chain of Hb. Given that rational, the number of HID2 molecule in the starting structure should not largely influence the MD simulations trajectories obtained. The convergence of the MD simulations was confirmed using RMSD of Cα atoms of the protein (Figure S6). Three independent MD trajectories lasting 50–70 ns were employed for the analysis. To further clarify the contribution of the residues evaluated by SPR analysis to the HID2-Hb interaction, the distances between pairs of atoms on HID2 and Hb were computed as frequency histograms. The following pairs of atoms were examined: HID2 Arg196 (NE)-heme (O1D), HID2 Tyr197 (OH)-heme (O1A or O2A), HID2 Gln209 (NE2)-Hb His45 (CB), HID2 Ile224 (CG2)-Hb Lys60 (CB), HID2 Ser225 (OG)-Hb Asp64 (OD2) and HID2 Met238 (CB)-Hb Leu86 (CD2), all of which participate in the interactions at the interface of HID2-Hb. As shown in Fig. 4, the histograms of distances representing hydrogen bonds in which Tyr197 or Ser225 of HID2 participate, showed sharp peaks at 2.6 Å, suggesting that these hydrogen bonds strongly contribute to the HID2-Hb interaction during most of the simulation time. The salt bridge in which Arg196 participates, and the van der Waals contacts involving Ile224 and Met238, showed a robust peak at a reasonable distance for each class of interaction. The broader peak observed for Met238 when compared to that of Ile224 may reflect the greater conformational space of the larger side-chain of the methionine residue. In contrast, the histogram for Gln209 did not show the characteristic shape consistent with a stable interaction with Hb, indicating a limited contribution to the interaction with the α-chain of Hb. This analysis is consistent with the results from SPR. Moreover, MD simulations revealed that Tyr197 and Ser225 had the most critical effect among four key residues identified by SPR analysis.

### Potential routes to inhibit HID2-Hb interactions

**Figure 5:**
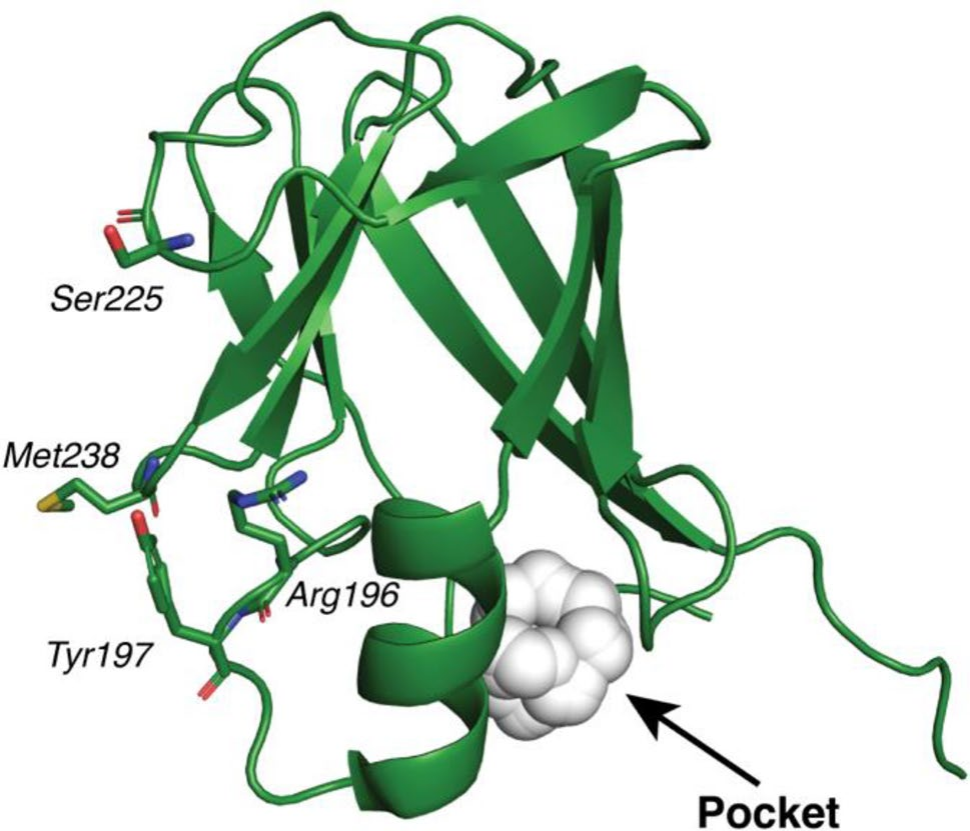
A cavity on HID2. The cavity with a volume of 139 Å^3^ is shown. HID2 in the unbound form was used as an input structure in identification of the cavity by PyVOL.

The strong effect of each and every mutation in the affinity for Hb indicated that essentially all components at the interface are critical for binding. The little tolerance of HID2 to mutations at the binding interface with Hb may be explained by the small interaction surface area between HID2 and Hb. The high sensitivity of this interface to changes in its chemical environment suggest that small molecules binding to that interfere or its immediate vicinity could interfere with or even block the binding of HID2 to Hb. Indeed, using PyVOL^19^, a few cavities with different sizes were identified in the proximity of the interface of HID2 with Hb. However, only one of these cavities exhibited sufficiently large volume (139 Å^3^) to accommodate the binding of a small molecule (Fig. 5)^20^. Alternatively, antibodies recognizing the interface of HID2 could be a promising agent for the competitive impairment of this protein–protein interaction. Given that Shr is pathogenically important and that the protein–protein interaction between HID2 and Hb is the first step of iron-acquiring process by Shr, both small molecule and antibody could serve as potential routes to inhibit this important nutritional pathway and could act as candidate antimicrobial agents.

In summary, we report the crystals structure of the complex between the Hb-binding domain HBD2 of Shr of the human pathogen *S. pyogenes* and human Hb. The structure revealed that HID2 binds to the heme pocket region of Hb, ensuring that Shr only binds to heme-containing Hb. This structure has shown the initial step of heme acquisition by Shr, in which HID2 binds to Hb, but cannot suggest a detailed mechanism of heme removal from Hb. Also, the analysis of the binding interface gives some insight into the potential drug modalities to regulate this important protein–protein interaction system.

## Materials and methods

### Expression and purification of HID2

Recombinant HID2 (residues 169–294) was prepared using *E. coli* BL21(DE3) (Millipore) previously transformed with vector pET28b containing the sequence of HID2 and a His_6_-tag and TEV protease cleavage site at the N-terminal. *E. coli* cells were initially cultured in six mL of LB medium containing 50 µg/mL kanamycin at 28 ℃ for 16 h, and subsequently transferred to 1 L fresh LB medium containing the same concentration of kanamycin and cultured again at 28 °C until the O.D.600 value reached a value of 0.4. At that moment β-D-1-thiogalactopyranoside (0.5 mM) was added to induce expression of the recombinant protein and the temperature lowered to 20 ℃ for 16 h. After that period, *E. coli* cells were collected by centrifugation at 7000×*g* at 4 ℃ for 10 min. The cell pellet was resuspended with binding buffer (20 mM Tris, 500 mM NaCl, 5 mM Imidazole, pH 8.0), and sonicated with a TOMY UD-201 instrument at 4 ℃ for 10 min. The insoluble fraction was removed by centrifugation at 40,000 g at 4 ℃ for 30 min. After the eliminating the debris with 0.8 µm filter, the supernatant was subjected to immobilized-metal affinity chromatography using Ni–NTA agarose (QIAGEN) equilibrated with binding buffer (column volume (CV) = 1 mL). The His_6_-tagged protein was eluted in the presence of the same buffer supplemented with 300 mM imidazole and incubated at 4 ℃ for 16 h in the presence of 0.1 mg/mL TEV protease while dialyzing against a buffer composed of 20 mM Tris and 200 mM NaCl (pH 8.0). The digested tag was removed by a second nickel chromatography in which the protein obtained in the flow-through was further purified by size exclusion chromatography using a Hiload 26/60 Superdex-75 column (Cytiva) equilibrated with the SEC buffer (20 mM Tris, 200 mM NaCl pH 8.0). Monomeric protein appearing as the main elution peak was collected and concentrated to the desired level. The concentration of HID2 was determined by a spectrophotometric at 280 nm.

### Preparation of human hemoglobin (for in vitro assays)

For in vitro assays, human hemoglobin powder was purchased from Sigma Aldrich. A total of 50 mg were dissolved in PBS buffer and purified by gel filtration using a Hiload 26/60 Superdex-200 column (Cytiva). The concentration of human hemoglobin was determined using PierceTM BCA Protein Assay Kit (Thermo Fisher Scientific) following the manufacturer’s instructions.

### Circular dichroism

The CD spectrum of each protein sample was measured at 20 °C using JASCO J-1100 spectropolarimeter in a 1 mm path-length quartz cell. Protein was prepared at 0.2 mg/mL in 20 mM Tris, 200 mM NaCl, pH 8.0 buffer.

### Surface plasmon resonance

The interaction between HID2 and human Hb was measured with a Biacore 8 K instrument (Cytiva) at 25 ℃. HID2 was immobilized on the surface of a CM5 sensor chip by the method of amine coupling at pH 5.5 as previously described, yielding approximately 120 RU of immobilization response^21^. The running buffer was 10 mM HEPES, 150 mM NaCl, 1 mM dithiothreitol, and 0.005% Surfactant P-20 (pH 7.5). Human Hb purified based on the method above and matched to the running buffer by dialysis was injected to the sensor chip surface at 30 µL/min in concentration series from 0.625 to 20 µM. The association time was 20 s and the dissociation time was 200 s. The *K*_*D*_ value was calculated from the individual binding rate constants (*k*_*on*_ and *k*_*off*_) using the BiaEvaluation software implemented in the instrument.

### Preparation of HID2-Hb complex for crystallography

Human hemoglobin was purified from a sample of blood collected from a single healthy donor, from which informed consent was obtained. All methods were carried out in accordance with relevant guidelines and regulations. The methodology was approved by the relevant committee of the Hospital of the Institute of Medical Science at the University of Tokyo. Briefly, heparin-treated blood was centrifuged and washed with saline buffer, followed by treatment with CO to produce the carboxyhemoglobin (COHb) form^22^. The cell suspension was incubated on ice in a low ionic strength buffer composed of 50 mM Tris (pH 8.6) and 2 mM EDTA to induce hemolysis. Debris were separated by centrifugation and COHb was purified by anion exchange chromatography using a Resource Q column. The purified hemoglobin was concentrated to 100 mg/ml using Amicon filters (Merck Millipore). To prepare the complex with HID2, human Hb and HID2 were mixed at a molar ratio of 1 to 1.1 and incubated at room temperature for 10 min. Subsequently, the complex was purified by size exclusion chromatography using a Superdex 75 10/300 column (Cytiva) equilibrated with 20 mM Tris–HCl and 200 mM NaCl at pH 8.0.

### Crystallization, data collection and processing

Purified recombinant HID2 at 8.3 mg/mL was crystallized using the method of hanging drop in a solution containing 20% PEG-MME 2000 (20% w/v), and 150 mM KBr, and 20 mM TRIS–HCl (pH 8.0). The complex of HID2 with human Hb was crystallized by the method of hanging drop in a solution composed of 23% PEG 3350 (w/v), 100 mM ammonium sulfate, and 100 mM BIS–TRIS (pH 5.5). The long crystallization process was conducive to the loss of the CO ligand from the Hb molecule. Suitable crystals were harvested, briefly incubated in mother liquor supplemented with 15% glycerol (crystals of HID2 alone) or 25% glycerol (complex with Hb) and transferred to liquid nitrogen for storage. Diffraction data were collected in beamline AR-NE3A (crystals of HID alone) and AR-NW12A (HID2-Hb complex) at the Photon Factory (Tsukuba, Japan) under cryogenic conditions (100 K). Diffraction images were processed with the program MOSFLM and merged and scaled with the program SCALA and AIMLESS^23^ of the CCP4 suite^24^. The structure of HID2 and HID2 in complex with Hb were determined by the molecular replacement method with PDB entry code 6DKQ^11^ and 4NI0^25^ and with the program PHASER^26^. The models were refined with the programs REFMAC5^27^ and built manually with COOT^28^. Validation was carried out with PROCHECK^29^. Data collection and structure refinement statistics are given in Table 2.

### Molecular dynamics (MD) simulations

GROMACS 2016.332^30^ with the CHARMM36m force field^31^ was employed for MD simulations of the HID2-Hb complex. For solvation of the protein complex, TIP3P^32^ water molecules were placed in a rectangular box such that the minimum distance to the edge of the box was 15 Å under periodic boundary conditions. Through the CHARMM-GUI, 150 mM of Na^+^ and Cl^-^ ions were added to imitate a salt solution. Before the MD simulation, energy minimization for 5000 steps and equilibration with the NVT ensemble (303 K) for 1 ns were conducted. Simulations were performed with the NPT ensemble at 303 K. The time step was set to 2 fs, and snapshots were saved every 10 ps throughout the simulations. The cutoff distances for Coulomb and van der Waals interactions were set to 12 Å. The particle mesh Ewald method^33^ was used for the evaluation of long-range electrostatic interactions, whereas the LINCS algorithm^34^ was used to constrain covalent bonds involving hydrogen atoms. For the calculation of the distance between the atoms, converged trajectories were analyzed using GROMACS.

### Supplementary Information

Supplementary Information.

## Data Availability

The coordinates and structure factors of unbound HID2 (entry code 7CUD) and HID2 in complex with Hb (entry code 7CUE), have been deposited in the Protein Data Bank.
