# Supplementary material for: Structural basis for the recognition of human hemoglobin by the heme-acquisition protein Shr from Streptococcus pyogenes
Source: Sci Rep. 2024 Mar 5;14:5374. doi: 10.1038/s41598-024-55734-x (PMC10912661; doi:10.1038/s41598-024-55734-x)
Supplement: Supplementary file 1 — Supplementary Information. [file 41598_2024_55734_MOESM1_ESM.pdf]

Structural basis for the recognition of human hemoglobin by the heme-acquisition protein Shr from *Streptococcus pyogenes*.

Akinobu Senoo[1,2], Masato Hoshino[3], Toshiki Shiomi[1], Makoto Nakakido[2], Satoru Nagatoishi[4], Daisuke Kuroda[5], Ichiro Nakagawa[6], Jeremy R.H. Tame[7], Jose M.M. Caaveiro[1,3,*], and Kouhei Tsumoto[2,3,8,*]

[1]*Laboratory of Protein Drug Discovery, Graduate School of Pharmaceutical Sciences, Kyushu University, 3-1-1 Maidashi, Higashi-ku, Fukuoka City, 812-8582 Japan.*

[2]*Department of Chemistry and Biotechnology, School of Engineering, The University of Tokyo, 7-3-1 Hongo, Bunkyo-ku, Tokyo, 113-8656 Japan.*

[3]*Department of Bioengineering, School of Engineering, The University of Tokyo, 7-3-1 Hongo, Bunkyo-ku, Tokyo, 113-8656 Japan.*

[4]*Medical Device Development and Regulation Research Center School of Engineering, The University of Tokyo, 7-3-1 Hongo, Bunkyo-ku, Tokyo, 113-8656 Japan.*

[5]*Research Center for Drug and Vaccine Development, National Institute of Infectious Diseases, 1-23-1 Toyama, Shinjuku-ku, Tokyo 162-8640, Japan.*

[6]*Department of Microbiology, Graduate School of Medicine, Kyoto University, Yoshida-Konoe-cho, Sakyo-ku, Kyoto 606-8501, Japan.*

[7]*Drug Design Laboratory, Graduate School of Medical Life Science, Yokohama City University, 1-7-29 Suehiro, Yokohama, Kanagawa, 230-0045 Japan.*

[8]*The Institute of Medical Sciences, The University of Tokyo, 4-6-1 Shirokanedai, Minato-ku, Tokyo, 108-8629, Japan.*

Table S1: Protein-protein interaction surface.

**Interface with α-chain**[a]		**Interface with β-chain**[b]	
**Residue**[b]	**BSA (Å^2)**	**Residue**[b]	**BSA (Å^2)**
Hemoglobin		***Hemoglobin***	
His 45	78.1	Ser 44	44.9
Gly 57	23.9	Lys 59	48.5
Lys 60	54.8	Ala 62	25.8
Lys 61	84.0	Lys 65	33.6
Asp 64	23.9	Lys 66	77.6
Ala 82	25.3	Thr 87	25.6
Leu 83	15.1	Leu 88	16.1
Leu 86	45.6	Leu 91	45.7
Lys 90	47.8	Lys95	65.2
Leu 91	19.2	Leu 96	22.8
Others	20.1	Others	12.8
Subtotal	*438*	*Subtotal*	*419*
HID2		***HID2***	
Arg 196	32.9	Arg 196	28.1
Tyr 197	83.2	Tyr 197	81.1
Gln 208	52.0	Gln 208	47.3
Gln 209	16.3	Gln 209	17.9
Ile 224	81.1	Ile 224	88.6
Ser 225	49.1	Ser 225	39.1
Asp 226	30.0	Met 238	94.0
Met 238	99.7	Lys 260	23.0
Others	22.0	Others	12.8
Subtotal	*466*	*Subtotal*	*466*
Total	***904***	***Total***	***885***

[a]Corresponds to one complex.
[b]Corresponds to the average of two complexes.

Table S2: Polar interactions between Hb and HID2

PDB ID 7CUE (This study)

**Protein-protein interaction**		
**Chain A (Hb α-chain) and Chain E (HID2)**		
**Residue from Hb**	**Type**	**Residue from HID2**
Asp64	HB	Ser225
Lys60	SB	Asp226
**Chain B (Hb β-chain) and Chain F (HID2)**		
**Residue from Hb**	**Type**	**Residue from HID2**
Ser44	HB	Gln209
**Chain D (Hb β-chain) and Chain H (HID2)**		
**Residue from Hb**	**Type**	**Residue from HID2**
Lys95	SB	Asp195

**Interactions at heme-protein interface**		
**Heme A and Chain E (HID2)**		
**Residue from Hb**	**Type**	**Residue from HID2**
HEM	HB	Arg196 (NE)
HEM	HB	Arg196 (NH1)
HEM	HB	Tyr197
HEM	HB	Met238
**Heme B and Chain F (HID2)**		
**Residue from Hb**	**Type**	**Residue from HID2**
HEM	HB	Arg196
HEM	HB	Tyr197
**Heme D and Chain H (HID2)**		
**Residue from Hb**	**Type**	**Residue from HID2**
HEM	HB	Arg196
HEM	HB	Tyr197

PDB ID 8DOV (PNAS)

**Protein-protein interaction**		
**Chain A (Hb α-chain) and Chain J (HID2)**		
**Residue from Hb**	**Type**	**Residue from HID2**
Asp64	HB	Ser225
Lys60	HB, SB	Asp226
**Chain B (Hb β-chain) and Chain I (HID2)**		
**Residue from Hb**	**Type**	**Residue from HID2**
Lys95	HB	Asp199
**Chain D (Hb β-chain) and Chain K (HID2)**		
**Residue from Hb**	**Type**	**Residue from HID2**
Lys95	HB	Tyr197
Lys95	SB	Asp195

**Interactions at heme-protein interface**		
**Heme A and Chain E (HID2)**		
**Residue from Hb**	**Type**	**Residue from HID2**
HEM	HB	Arg196
HEM	HB	Tyr197
HEM	HB	Met238
**Heme B and Chain I (HID2)**		
**Residue from Hb**	**Type**	**Residue from HID2**
HEM	HB	Arg196
HEM	HB	Tyr197
HEM	HB	Met238
**Heme D and Chain K (HID2)**		
**Residue from Hb**	**Type**	**Residue from HID2**
HEM	HB	Arg196
HEM	HB	Tyr197
HEM	HB	Met238

HB: hydrogen bond, SB: salt bridge, n.d.: not detected

Table S3: Protein-heme interaction surface.

**Interface with α-chain**[a]		**Interface with β-chain**[b]	
**Residue**[b]	**BSA (Å^2)**	**Residue**[b]	**BSA (Å^2)**
Hemoglobin		***Hemoglobin***	
Heme 202	156	Heme 202	164
Subtotal	*156*	*Subtotal*	*164*
HID2		***HID2***	
Arg 196	24.5	Arg 196	22.9
Tyr 197	55.4	Tyr 197	53.1
Asn 237	14.7	Asn 237	15.7
Met 238	18.9	Met 238	19.6
Others	10.1	Others	10.6
Subtotal	*124*	*Subtotal*	*122*
Total	***280***	***Total***	***286***

[a]Corresponds to one complex.
[b]Corresponds to the average of two complexes.

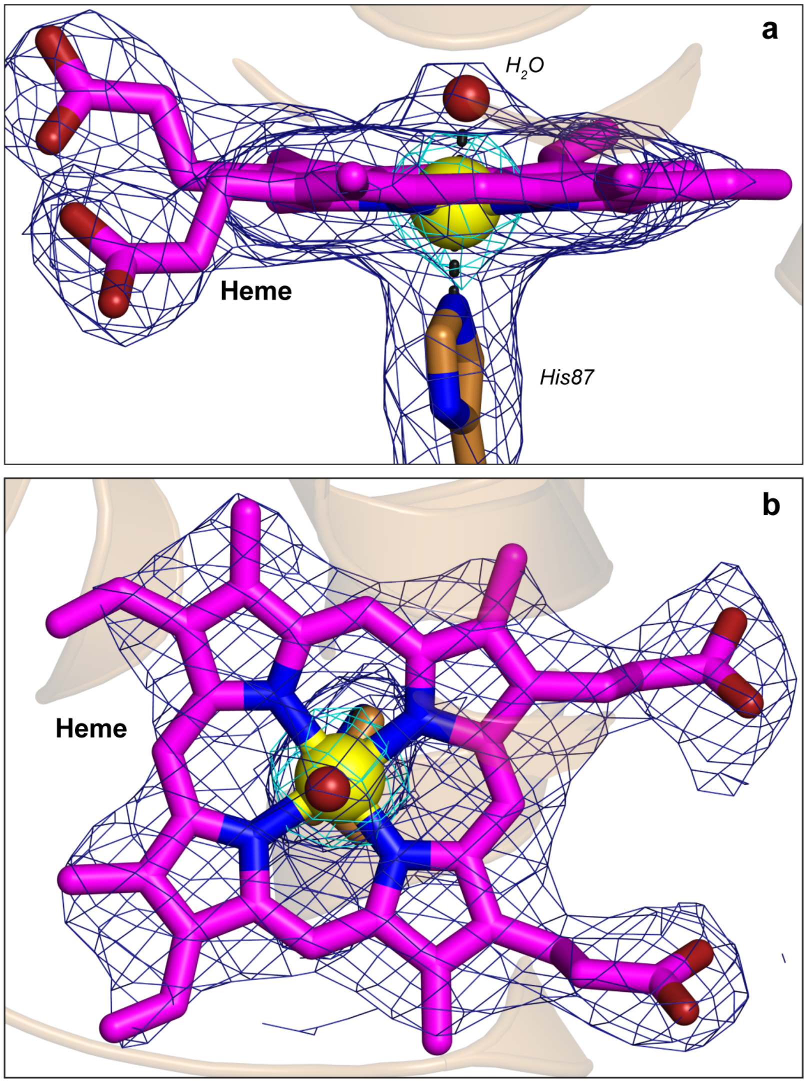

Figure S1. Electron density of heme bound to Hb in the presence of HID2. Lateral **(a)** and top **(b)** views of heme and the corresponding SigmaA weighted 2Fo − Fc electron density including the axial ligand His87 (belonging to the α-chain of human Hb) and a distal water molecule. The map is contoured at a σ level of 1.0 (blue) and 6.0 (cyan). The heme group is shown as magenta sticks, and its iron atom is depicted as a yellow sphere. Axial ligands His87 (proximal) and a water molecule (distal) are shown as brown sticks and a red sphere, respectively.

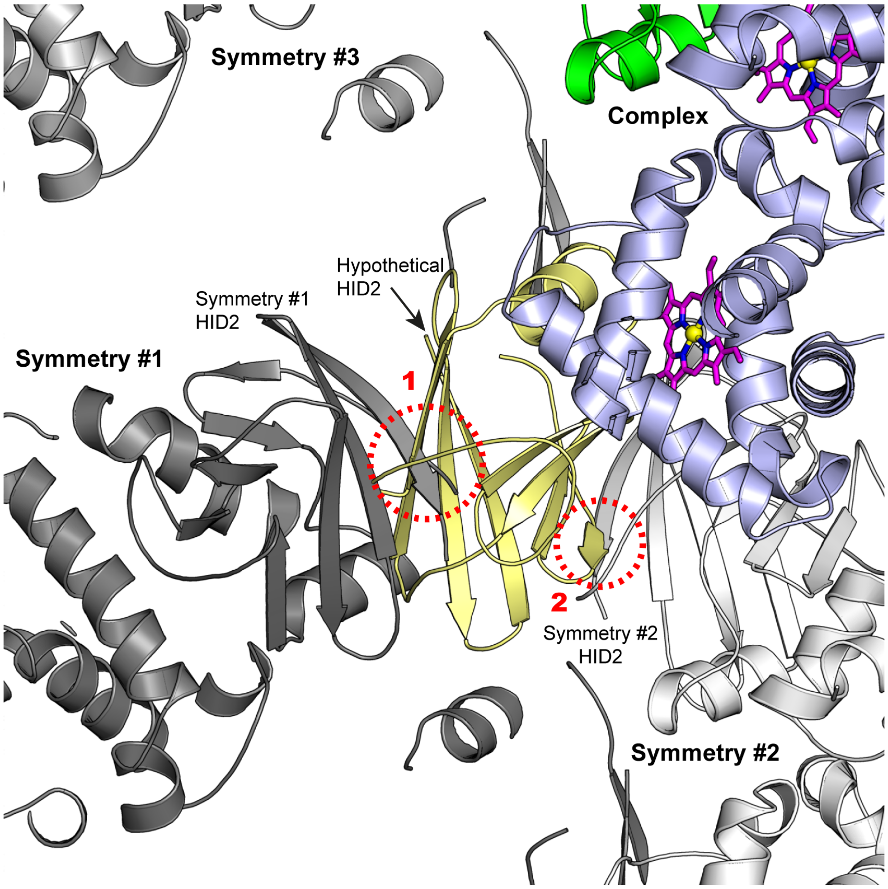

Figure S2. Steric hindrance of a hypothetical fourth molecule of HID2 in the asymmetric unit. In this figure, a hypothetical fourth molecule of HID2 (yellow) is placed in the binding interface of Hb, within the crystal lattice, and thus including symmetry related protein chains. In such a scenario, the hypothetical molecule of HID2 would clash with symmetry mates in region #1 and in region #2 (both indicated by red dashed circles). This model explains why a fourth molecule of HID2 cannot be bound to Hb in this crystal lattice.

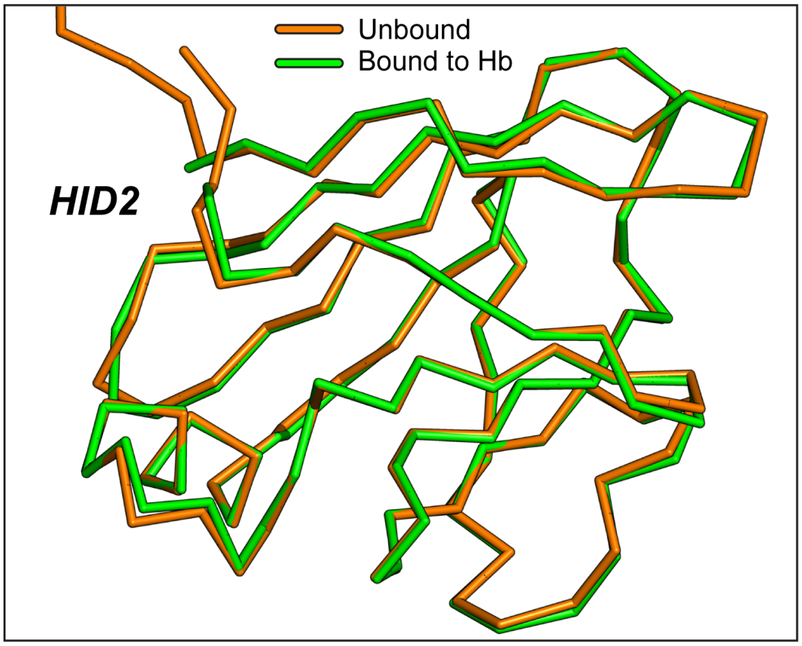

Figure S3. Similarity between bound and unbound HID2. Superposition of the crystals structures of HID2 bound to human Hb (green) and HID2 in the unbound form (orange). The average RMSD achieved between the three chains in the asymmetric of the complex and the two chains in the asymmetric unit of unbound HID2 was 0.6 Å.

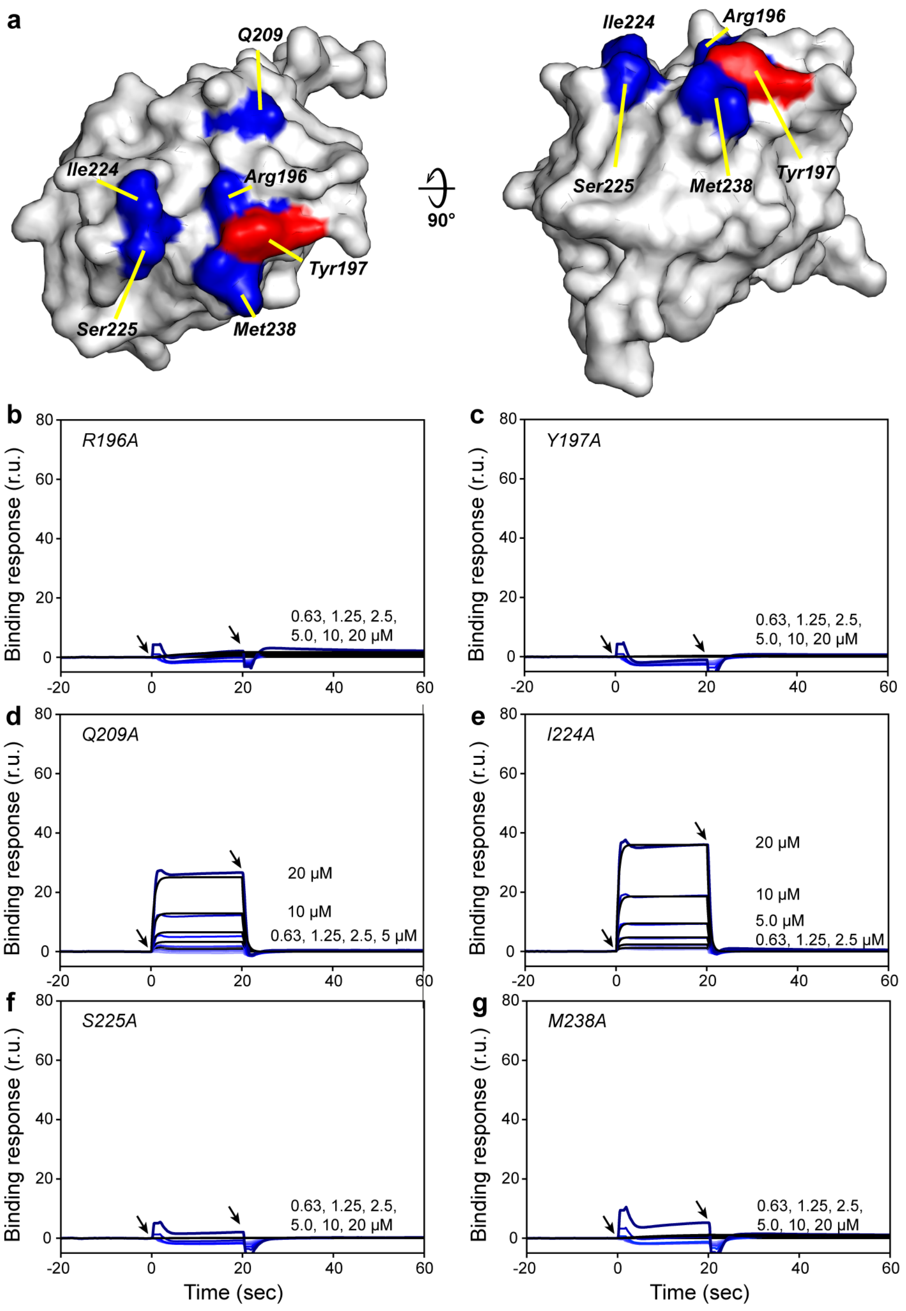

Figure S4. Mutational analysis. (a) Location of the residues mutated on the surface of HID2. Sensorgrams of binding of mutants of HID2 **(b)** R196A, **(c)** Y197A, **(d)** Q209A, **(e)** I224A, **(f)** S225A, and **(g)** M238A to human Hb are shown. Kinetic data are shown in blue and fitting curves calculated by BiaEvaluation in black. Binding was not detected in mutants R196A, Y197A, S225A, and M238A. All the mutated residues of HID2 are depicted in Figure 2c. The ordinate scale is the same as that in Figure 1 of the main text.

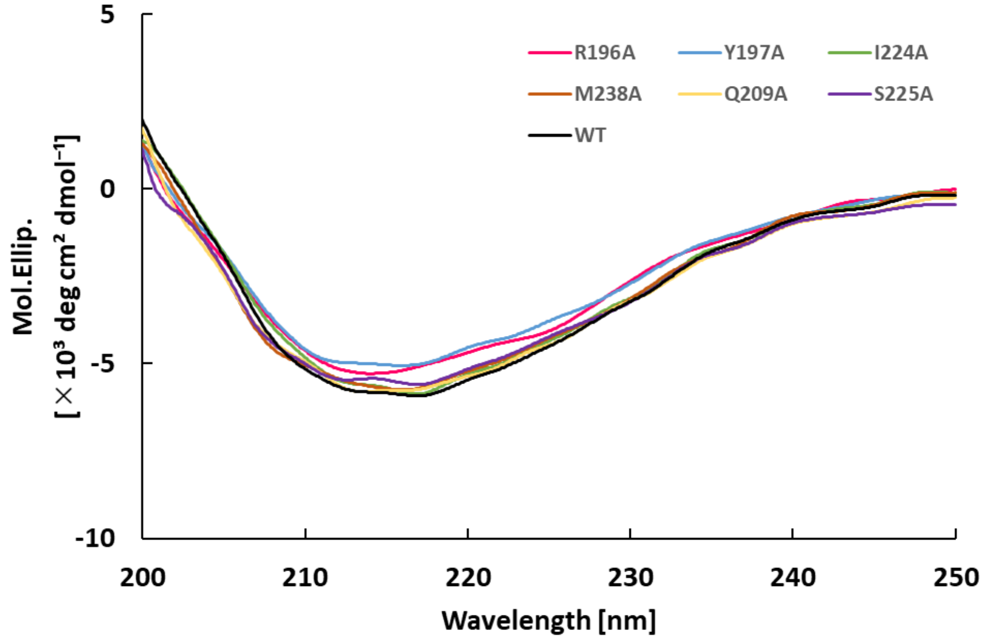

Figure S5. Circular dichroism (CD) spectra of HID2 and several of its mutants.

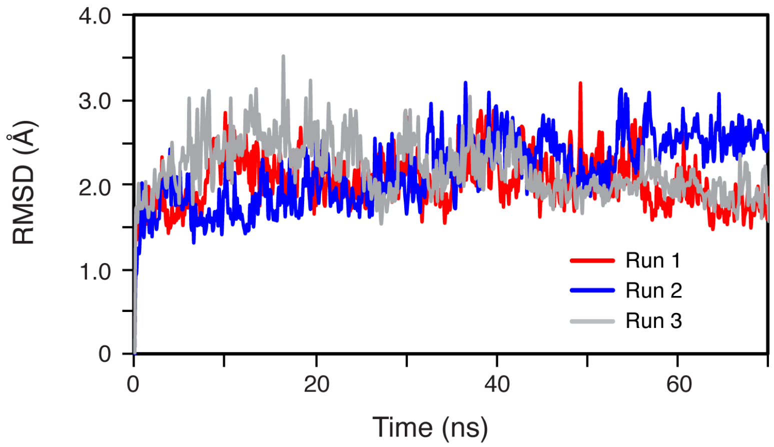

Figure S6 MD simulations of the HID2-Hb complex. The panel shows the root mean square deviation (RMSD) of Cα atoms in three independent MD trajectories.
